# Implementation and evaluation of adverse drug reaction monitoring system in a tertiary care teaching hospital in Mumbai, India

**DOI:** 10.2478/intox-2013-0008

**Published:** 2013-03

**Authors:** Dindayal Patidar, Mithun S. Rajput, Nilesh P. Nirmal, Wenny Savitri

**Affiliations:** 1 Department of Pharmacology, Indore Institute of Pharmacy, SGI, Pithampur Road, Rau, Indore-453 331, India; 2 College of Pharmacy, IPS Academy, Rajendra Nagar, A.B. Road, Indore-452 012, India; 3 Faculty of Pharmaceutical Sciences, Prince of Songkla University, Hat Yai-90112, Songkla, Thailand; 4 Faculty of Nursing, Prince of Songkla University, Hat Yai-90112, Songkla, Thailand

**Keywords:** adverse drug reaction, hospital based monitoring, pharmacovigilance, questionnaire survey

## Abstract

Adverse drug reactions (ADR) are a significant cause of morbidity and mortality, often identified only post-marketingly. Improvement in current ADR reporting, including utility of underused or innovative methods, is crucial to improve patient safety and public health. Hospital-based monitoring is one of the methods used to collect data about drug prescriptions and adverse events. The aims of this study were to identify the most frequent ADRs recognized by the attending physicians, study their nature, and to target these ADRs in order to take future preventive measures. A prospective study was conducted over a 7-month period in an internal medicine department using stimulated spontaneous reporting for identifying ADRs. Out of the 254 admissions, 32 ADRs in 37 patients (14.56%) were validated from the total of 36 suspected ADRs in 41 patients. Female predominance was noted over males in case of ADRs. Fifty percent of total ADRs occurred due to multiple drug therapy. Dermatological ADRs were found to be the most frequent (68.75%), followed by respiratory, central nervous system and gastrointestinal ADRs. The drugs most frequently involved were antibiotics, anti-tubercular agents, antigout agents, and NSAIDs. The most commonly reported reactions were itching and rashes. Out of the 32 reported ADRs, 50% of the reactions were probable, 46.87% of the reactions were possible and 3.12% of the reactions were definite. The severity assessment done by using the Hartwig and Seigel scale indicated that the majority of ADRs were ‘Mild’ followed by ‘Moderate’ and ‘Severe’ reactions, respectively. Out of all, 75% of ADRs were recovered. The most potent management of ADRs was found to be drug withdrawal. Our study indicated that hospital based monitoring was a good method to detect links between drug exposure and adverse drug reactions. Adequate training regarding pharmacology and optimization of drug therapy might be helpful to reduce ADR morbidity and mortality.

## Introduction

Drug event monitoring is a method of active pharmacovigilance surveillance. Schemes for spontaneous reporting of suspected adverse drug reactions (ADRs) have an important role in identifying such effects which were not found in pre-marketing trials. In many instances, regulatory and public health decisions have to be made on the basis of data from spontaneous reports (MCA, 2000). Although such schemes are useful to safeguard public health, they have several weaknesses, including underreporting (Eland *et al.,* 1999). Active surveillance can be achieved by reviewing medical records or interviewing patients and/or physicians to ensure complete and accurate data on adverse events. Hospital based monitoring is one of the systems used to collect data on drug prescriptions and adverse events. In this approach, trained health personnel monitor patients, admitted to selected hospitals by reviewing their clinical charts and conducting structured interviews of both patients and physicians. Information on patient demographics, indication for treatment, duration of therapy, dosage, clinical events and reasons for discontinuation can be included in the questionnaire. Such projects have proved useful for the study of acute and relatively common ADRs (Schumock *et al.,* 1995; Levy *et al.,* 1999; Van Puijenbroek *et al.,* 2002; Coulter, 2002). ADRs and events have a considerable impact not only on the health of the population but also on health care costs; they account for 5% of all hospital admissions, occur in 10–20% of inpatients, cause death in 0.1% of medical and 0.01% of surgical inpatients and increase the costs of patient care (Meyboom *et al.,* 2002; Pirmohamed *et al.,* 1998).

The presence of ADRs may be underestimated in part because treating physicians fail to recognize ADRs, as they tend to mimic any naturally occurring disease process, by acting through the same physiological and pathological pathways. A study demonstrated that up to 57% of the community acquired adverse drug reactions are not recognized by the attending physician upon hospital admission, leading to inappropriate management of the adverse event, exposure of the patient to additional ADRs of the drugs and prolonged hospitalization (Dormann *et al.,* 2003). The primary purposes of the present prospective study were: (1) to characterize the nature, early detection, severity and preventability of the ADRs; (2) to describe the drugs most frequently involved in adverse reactions and the main predisposing factors leading to ADRs and (3) to estimate the incidence of ADRs in hospitalized patients. The secondary objectives include (1) implementation of regulatory action to maximize the benefit and minimize the risks associated with medicinal products; (2) to determine the significance of ADRs that were reported by patients but unknown to their provider; (3) to convince healthcare professionals that reporting of ADR is their professional and moral obligation, and (4) to anticipate the various combinations by which ADRs can be caused.

## Subjects and methods

This study was a concurrent, spontaneous reporting, involving both active and passive methods. Active methods include physicians, pharmacists and nurses actively looking for suspected ADRs and passive methods include stimulating prescribers to report suspected ADRs. The study was conducted in a 35-bed internal medicine ward of the Holy Family Hospital, Bandra (W), Mumbai, India, over a period of 7 consecutive months, starting from July 2008 to January 2009. The study protocol was reviewed and approved by the University Ethics Committee.

All the physicians in the ward were informed about the study, outlining the ADRs’ negative impact and were asked to report all observed adverse events. In order to ensure that the rate of notifications remains constant during the whole study period, the physicians were regularly reminded about the study taking place.

An Adverse Drug Reaction Reporting Form was designed and made available at all nursing stations of the ward of the hospital for easy access to all healthcare professionals. The Adverse Drug Reaction Reporting Form was prepared with reference to the ADR reporting form of the Central Drug Standard Control Organization (CDSCO) which includes information about the patient, like name, age, sex, medication history, diagnosis history, name of the suspected drug along with batch number, lot number manufacturing date and expiry date. The route of drug administration, frequency and dose is also mentioned in the form. Basic information of adverse reaction caused by the suspected drug was also included. We defined adverse drug reactions according to the World Health Organization definition, as being all “noxious and unintended drug response, which occur at doses normally used in man for prophylaxis, diagnosis, or therapy of disease or for the modification of physiological function (WHO, 1972). By this definition, ADRs primarily include allergic reactions and adverse effects. Therefore, we excluded all the intentional overdoses, poisonings and therapeutic failures.

In addition, the patient's medication history was also taken and any co-morbidity identified to assess the causality relationship between the suspected drug and reaction. Patients who developed an ADR were interviewed daily from the day the ADR was reported with regard to consumption of any other medication. The relationship between ADR and the suspected drug was assessed. The severity of the ADRs was also assessed in different categories as mild, moderate and severe for each ADR. All the reported ADRs were assessed for their preventability criteria. Personalized letters and circulars signed by the director of the hospital were circulated to all residents and practitioners, visiting practitioners and nursing stations. These letters contained information on the number of suspected ADRs that had been reported till date, need for continuing reporting of ADRs and a request to maintain a high degree of suspicion for the ADRs. The data observed were analyzed in order to study the characteristics of the ADRs and to determine the nature and pattern of ADRs related to hospital admission and difference in the severity of ADRs and management and outcome of management of the reported ADRs. Causality assessment is the method by which the extent of relationship between a drug and a suspected reaction is established. The assessment of causality relationship is often subjective, based upon an individual clinician's assessment. One clinician's judgement may appear unlikely to another clinician. If an ADR is suspected, the assessment starts with collection of all the relevant data pertaining to patient demographics, medications, including non-prescription (OTC) drugs, comprehensive ADR details including a description of the reaction, time of onset and duration of the reaction, complications and/or sequelae treatment of the reaction and outcome of the treatment and further relevant investigation reports. The collected data were used to correlate and categorize the relationship between the suspected drug and the adverse drug reaction. Causality assessment was done using the Naranjo's scale (Naranjo *et al.,* 1992; Naranjo *et al.,* 1981). The data were also analyzed as per severity (Mild, Moderate and Severe) of the suspected adverse drug reaction (Hartwig *et al.,* 1992) and categories as death, life threatening, hospitalization (initial or prolonged), disability, congenital anomaly, required intervention to prevent permanent impairment or damage, not serious, and others.

## Results

During the study period, 254 patients (128 males and 126 females) with an average of 36.28 per month were admitted to the ward with planned and non-planned admissions. The average length of stay was 1.03 days. The average patient age was 50.8 years. Eighteen patients were found to be habitual smokers, including 1 female, and 26 patients were found to be alcoholics, including 4 females. A total of 9 patients were found to be habitual of both alcohol and smoking including 1 female (Table 1).

**Table 1:** Distribution of patient pool as per their social habits.

Habits	No. of patients	Male	Female	Percentage
Smoking	18	17	1	7.08
Alcohol	26	22	4	10.23
Smoking + Alcohol	9	8	1	3.54
None	201	81	120	79.13
Total	254	128	126	99.98

During the 7 months of the study period including 254 patients, the physicians reported 36 suspected ADRs in 41 patients and 32 ADRs were validated in 37 patients (14.56% of the admitted patients). The ADRs that were not validated were unlikely according to the causality assessment. Some patients had several ADRs simultaneously or successively. Of the 254 hospitalized patients, the 32 ADRs represented an overall rate of 12.59%. Female predominance was noted over males in cases of ADRs. From the total number of patients with ADRs, 15 (45.94%) were men and 20 (54.05%) were women. Table 2 describes the patient pool as per sex and occurrence of ADRs.

**Table 2:** Distribution of patient pool as per sex.

	Admitted patients		Suspected ADRs			Validated ADRs		
Group	No.	%	No. of ADRs	Patients with ADRs	Patients with ADRs (%)	No. of ADRs	Patients with ADRs	Patients with ADRs (%)
Male	128	50.39	17	19	14.84	15	17	13.28
Female	126	49.61	19	22	17.46	17	20	15.87

All validated ADRs were classified as per age group. Patients in the age group of 61–80 years showed the highest number of ADRs, *i.e.* 10 (31.25%) (Table 3).

**Table 3:** Distribution of patient pool as per age.

	Patients		ADRs	
Group	No.	Percentage	No.	Percentage
0–20	11	04.33	1	03.12
21–40	49	19.29	9	28.12
41–60	71	27.95	7	21.87
61–80	97	38.18	10	31.25
81 and above	26	10.23	5	15.62

The majority of the patients who developed an ADR received more than 5–6 drugs at the time of experiencing an ADR. Out of the 32 reported reactions, 16 (50%) ADRs occurred due to multiple drug therapy, 7 (21.87%) ADRs were due to an inter-current disease, and 4 (12.50%) ADRs were due to previous exposure of the drug. In 5 (15.62%) reported ADRs no predisposing factors were involved.

Dermatological ADRs were the most frequent (68.75%), followed by respiratory, central nervous system (9.37% each), and gastrointestinal ADRs (6.25%). Hematological and cardiovascular system related ADRs were relatively few, with 3.12% each.

The drug class most commonly implicated with ADRs was antibiotics followed by anti-tubercular drugs, antigout drugs, NSAIDs and blood related products. The drug classes least affected were antiepileptic drugs, antiasthmatic drugs, antiemetic drugs, anticoagulants and antiplatelet drugs. The most commonly reported reactions were itching 11 (34.37%) cases, rashes 7 (21.87%) cases, giddiness 3 (9.37%) cases, breathlessness 2 (6.25%) cases and shivering 2 (6.25%) cases. Other reactions included hypotension, hematuria, urticaria, pruritis, gastritis, Steven Johnson Syndrome, drowsiness. The suspected ADRs belonged to categories of “probable” followed by “possible” in their causality relationship. Out of the 32 reported ADRs, 16 (50%) of the reactions were probable, 15 (46.87%) of the reactions were possible and 1 (3.12%) of the reactions was definite. Out of the 32 reported ADRs, 24 (75.00%) recovered, in 4 (12.50%) of the ADR patients the symptoms continued (discharged against medical advice), 3 (9.37%) were fatal, and 1 (3.12%) unknown. All the reported ADRs, their symptoms and outcome of management are summarized in Table 4.

**Table 4:** Drug caused ADRs, their symptoms and outcome of management.

Category of drug	No. of ADRs	Suspected reaction	%	Outcome
Antibiotics	13		40.62	
Ceftriaxone Azithromycin Metronidazole Ofloxacin Ciprofloxacin Amoxicillin	4 2 1 3 1 2	Giddiness (1), itching and rashes (3) Hypotension (1), Itching over body (1) Rashes (1) Itching all over the body (2) Shivering (1) Itching (1) Urticaria (1), rashes (1)		Recovered Recovered Recovered Fatal Continuing Recovered Recovered
Antitubercular	4		12.5	
Combination of rifampicin, pyrazinamide, ethambutol, isoniazid Pyrazinamide	3 1	Steven Johnson Syndrome (1), Gastritis (1), Rashes (1), Itching (1)		Fatal Fatal Recovered Continuing
Antigout	3		9.37	
Allopurinol	3	Skin rashes (1), Itching (1)		Continuing Recovered
Antiepileptic	2		6.25	
Fosphenytoin	2	Pruritis and dermatitis (1), itching (1)		Recovered
Blood related Products	2		6.25	
Blood transfusion	2	Breathlessness (1), Itching over body (1)		Recovered Recovered
Analgesics	2		6.25	
Tramadol	2	Drowsiness (1), Sweating and giddiness (1)		Recovered Recovered
NSAIDs	1		3.12	
Acetaminophen	1	Itching over palms (1)		Recovered
Antiasthamatic	1		3.12	
Ipratropium bromide	1	Itching and rashes (1)		Recovered
Antimalarial	1		3.12	
Artesunate	1	Shivering (1)		Recovered
Antiplatelet and anticoagulant	1		3.12	
Enoxaparin and Aspirin	1	Hematuria (1)		Continuing
Antiemetic	1		3.12	
Ondansetron	1	Breathlessness (1)		Recovered
Antidiuretics	1		3.12	
Vasopressin	1	Breathlessness (1)		Unknown

The severity assessment was done by using the Hartwig and Seigel scale. According to this ADR severity assessment scale, the level of severity of ADRs is classified on a scale from 1 to 7. Level 1 and 2 indicate ‘Mild’, level 3, 4(a) and 4(b) are ‘Moderate’, level 5, 6 and 7 are ‘Severe’. Out of the 32 reported ADRs, ‘Mild’ reactions accounted for 43.75%, ‘Moderate’ reactions accounted for 31.25% and only 9.37% of the reactions were reported to be ‘Severe”. However, 15.62% of ADRs were not serious (Table 5).

**Table 5:** Level of severity of reported ADRs.

Severity	No. of ADRs	Percentage
Mild	14	43.75
Level 1	5	
Level 2	9	
Moderate	10	31.25
Level 3	1	
Level 4(a)	2	
Level 4(b)	6	
Severe	3	09.37
Level 5	1	
Level 6	1	
Level 7	1	
Others (not serious)	5	15.62

In 15 (46.87%) cases, the suspected drug was withdrawn while no change was made with the suspected drug in 2 (6.25%) of the cases, and the dose was altered in 1 (3.12%) case. Symptomatic treatment was required in 13 (40.62%) cases, while 1 (3.12%) of the cases required specific treatment (Table 6).

**Table 6:** Management of reported ADRs.

Management	No. of ADRs	Percentage
Drug withdrawal	15	46.87
Symptomatic treatment	13	40.62
No Change	2	6.25
Dose altered	1	3.12
Specific treatment	1	3.12

## Discussion

Implementation of an ADR reporting and monitoring system in the internal medicine ward of the Holy Family Hospital, Mumbai, was successfully achieved by distribution of circulars, display of posters, oral campaigns, formal speech and personal interaction related to the importance of reporting ADRs by health care professionals. The present study was initiated in order to study the nature of ADRs and to identify the most frequent ADRs recognized and reported by the attending physicians, using stimulated reporting. We also focused on assessing the incidence of ADRs in hospitalized patients.

A total of 254 cases were studied during the study period, including 128 (50.39%) males and 126 (49.61%) females. Of these 15 (46.87%) males and 17 (53.12%) females had validated ADRs. A total of 19 (7.43%) patients were already allergic to various drugs. A total of 1522 medications were prescribed to the 254 patients. The average number of medications per patient was found to be 5.99 ± 0.10. All reported ADRs were suspected in inpatients. In 4 inpatients, the reason for admission was found to be ADRs, and those were of the severe category.

Various studies have reported that the percentage of ADRs found was higher in adults and the geriatric population. The present study revealed a predominance adults (49.99%) over the geriatric (46.67%) and pediatric (3.12%) populations. This might be due to the fact that most adult patients received multiple drug therapy and also presented with other co-morbidities such as diabetes, hypertension, tuberculosis, and asthma. It is known that multiple drug therapy and co-morbidities predispose patients to adverse drug reactions. This finding is consistent with the results of the study carried out by Murphy *et al.* (1993) but differed from the study carried out by Lin and Lin (1991), who reported that drug related hospitalization was significantly higher in the geriatric population.

The most common category associated with ADRs was dermatology (68.75%). This finding is concurrent with the study carried out by Coelho *et al.* (2002) and Rajesh *et al.* (2008), but it differes from reports of Suh *et al.* (2000), where gastrointestinal manifestations had the highest rate. In our study, the gastrointestinal system was associated with 6.25% of ADRs, while the respiratory system and central nervous system were associated with 9.25% of ADR each. It is known from the literature that the dermatological reactions occur most commonly with antibiotics and anti-tubercular drugs. Of the dermatological reactions observed in hospital, itching (34.37%) and rashes (21.87%) were found to be the most common. In the study, the drug class most commonly implicated with ADRs was antibiotics with the highest percentage (61.93%) followed by antitubercular drugs (12.19%). This result is consistent with the study carried out by Murphy *et al.* (1993), Carnasos *et al.* (1974) and Rajesh *et al.* (2008) and differes from the studies by Bergman *et al.* (1981) who reported that cardiovascular drugs were are the most commonly associated drug class. This might be associated with the fact that antibiotics were the most commonly used class of drugs in this study.

To strengthen and further emphasize the validity of the findings of the study, causality assessment was done by using the Naranjo's scale. Out of the 32 ADRs reported, 15 (50%) ADRs were probable, 15 (46.87%) were possible and 1 (3.12%) were definite. On evaluation of the severity of ADRs by the Hartwig and Siegel severity assessment scale, it was evident that most of the ADRs reported in the study were of mild severity. Even though various incidences supported the finding that the most common ADRs were are skin reactions, there had been very little effort to curtail their severity. Reactions like Steven Johnson Syndrome pose a significant risk to the patient's life. This further emphasizes the importance of monitoring ADRs.

Worldwide studies have proved ADRs to be a major cause of morbidity and mortality. Though Indian studies in this regard are very few, the pattern of reactions seems to be similar. There are however certain peculiarities of drug use in our situation, such as: large number of patients, poor doctor-patient ratio, self-medication, and drugs of alternative systems of medicine, malnutrition, widespread anemias, presence of counterfeit drugs and presence of the highest number of drug combinational products in the world. The incidence of adverse drug reactions appears to be same as in the West or other countries. Unfortunately, in spite of the presence of five well organized centers for drug monitoring in the country, the number of reports sent annually is far from satisfactory. There are several reasons why the number of adverse drug reactions is so high. These include the high number of drugs prescribed are high, the ever-increasing number of new drugs in the market and the lack of a formal system for monitoring adverse drug reactions (Bates *et al.,* 1997).

While the exact epidemiology remains to be assessed in India, ADRs have recently emerged as leading killers. The management of drug-induced diseases requires more than 100 billion US dollars annually (Bremnan *et al.,* 1991). These astronomical figures are currently unmatched by the money involved in any single disease management. Nevertheless, several studies have shown that most ADRs are preventable, provided that the drugs are used rationally. However, the most common system failure has been to disseminate the knowledge of pharmacovigilance to the individuals actually involved in prescribing drugs, *i.e.* the physicians (Cohen, 1999). Principles and practice of pharmacovigilance seem to be more often discussed in an academic manner rather than in a pragmatic or applied sense. Such discussions are held among pharmacologists and pharmacists who are not directly involved in patient care, while physicians who treat cases and use drugs generally keep themselves uninvolved. Drug safety has been included in curriculum guidelines for Indian medical undergraduates (MCI Curriculum Guidelines, 1997) but little has been achieved in this regard (Leape, 1994).

Monitoring of adverse drug reactions should be a collaborative activity of both clinicians and pharmacologists. At present, in India, the pharmacologists usually do it with or without the involvement of clinicians (Uppal *et al.,* 2000). Physicians, however, continue to play a meaningful role in the entire monitoring process, as the co-operation of clinicians is needed to have access to patient data and interpretation of the reports of suspected adverse drug reactions. In many other countries, the pharmacists or nurses specially recruited for this purpose, carry out the task under supervision (Taylor *et al.,* 1994; Singh *et al.,* 1999). Physicians and pharmacologists are involved in the interpretation of the collected data or in hypothesis testing on the basis of the reports. These workers may be involved in a panel of the physicians in reviewing all the collected reports.

Most of the adverse drug reactions are preventable. This calls for the urgent need to reinforce the monitoring of adverse reactions to drugs, public education against self-medication, inclusion of reaction monitoring, and an introduction to drug-safety in the curriculum of medical undergraduates, as well as systemic and periodic medical education of health professionals. This multi-pronged strategy could lead to a reduction in the incidence of adverse drug reactions.

## Conclusion

The stimulated spontaneous reporting used in the present study turned out to be a pragmatic method which allowed the detection and characterization of ADRs. However, monitoring of adverse drug reactions is an ongoing, ceaseless and continuing process. Since newer and newer drugs hit the market, the need for pharmacovigilance grows more than ever before. Monitoring of the adverse effects of newer drugs, particularly of serious nature, is mandatory. Imparting knowledge and awareness of ADRs reporting among health care professionals would introduce the reporting culture among medical practitioners and increase the reporting rates of ADRs. Careful consideration involved in planning and monitoring of drug therapy will lead to prevention of ADRs. On balance, this study suggests that hospital-based monitoring is a good method to detect known and unknown links between drug exposure and ADRs.
